# Supplementary material for: FERN – a Java framework for stochastic simulation and evaluation of reaction networks
Source: BMC Bioinformatics. 2008 Aug 29;9:356. doi: 10.1186/1471-2105-9-356 (PMC2553347; doi:10.1186/1471-2105-9-356)
Supplement: Additional file 1 — FERN distribution, Version 1.3. This archive contains the FERN source code and binaries as well as documentation and example models in FernML and SBML. [file 1471-2105-9-356-S1.zip › fern/doc/javadoc/fern/simulation/algorithm/GillespieEnhanced.html]

GillespieEnhanced


---


|  |  |  |
| --- | --- | --- |
| **Overview** \| **Package** \| **Class** \| **Use** \| **Tree** \| **Deprecated** \| **Index** \| **Help** | |  |
| **PREV CLASS**   **NEXT CLASS** | **FRAMES**    **NO FRAMES**     **All Classes** |  |
| SUMMARY: NESTED \| FIELD \| CONSTR \| METHOD | DETAIL: FIELD \| CONSTR \| METHOD |  |


---


## fern.simulation.algorithm Class GillespieEnhanced

```
java.lang.Object
  fern.simulation.Simulator
      fern.simulation.algorithm.GillespieEnhanced
```

**Direct Known Subclasses:**: AbstractBaseTauLeaping

---

``` public class GillespieEnhanced extends Simulator ```

This is an enhanced version of the original Direct method developed by
Gillespie. Just like the algorithm of Gibson and Bruck it uses a dependency graph to know what propensities
have to be recalculated.

Take care with the option efficientlyAdaptSum: if it is true, it is possible that the sum
become numerically unstable and the simulation fails, but especially for sparse networks
it should be much more efficient.

For references see Daniel T. Gillespie., A General Method for Numerically Simulating
the Stochastic Time Evolution of Coupled Chemical Reactions, J.Comp.Phys. 22, 403 (1976)
and M.A.Gibson and J.Bruck, Efficient Exact Stochastic Simulation of Chemical
Systems with Many Species and Many Channels, J.Phys.Chem.A., Vol 104, no 9, 2000

**Author:**
:   Florian Erhard

**See Also:**: `GillespieSimple`, `DependencyGraph`

---

**Nested Class Summary**

**Nested classes/interfaces inherited from class fern.simulation.Simulator**

`Simulator.FireType`


| **Field Summary** | |
| --- | --- |
| `protected  double` | `a_sum` |

**Fields inherited from class fern.simulation.Simulator**

`a, stochastics, t`


**Constructor Summary**

`GillespieEnhanced(Network net)`


| **Method Summary** | |
| --- | --- |
| `protected  double` | `directMCTau(double sum)`             obtains a random (but following a specific distribution) timestep as described by the direct method in chapter 5A page 417ff |
| `String` | `getName()`             Gets the name of the algorithm. |
| `void` | `initialize()`             Initializes the algorithm: set t=0reset the `AmountManager`recalculate the propensities Gets called at the very beginning of `start` |
| `boolean` | `isEfficientlyAdaptSum()` |
| `void` | `performStep(SimulationController control)`             Performs one simulation step. |
| `void` | `reinitialize()`             Reset propensities when a event has been executed. |
| `void` | `setAmount(int species, long amount)`             Sets the amount of the given species. |
| `void` | `setEfficientlyAdaptSum(boolean efficientlyAdaptSum)` |
| `void` | `setVolume(double volume)`             Sets the volume of the reaction network. |

**Methods inherited from class fern.simulation.Simulator**

`addObserver, fireReaction, fireReaction, getAmount, getAmountManager, getNet, getNextThetaEvent, getPropensity, getPropensityCalculator, getTime, getVolume, isInterpolateTheta, registerNewTheta, setInterpolateTheta, start, start, thetaEvent`

**Methods inherited from class java.lang.Object**

`clone, equals, finalize, getClass, hashCode, notify, notifyAll, toString, wait, wait, wait`

**Field Detail**

### a\_sum

```
protected double a_sum
```


**Constructor Detail**

### GillespieEnhanced

```
public GillespieEnhanced(Network net)
```


**Method Detail**

### initialize

```
public void initialize()
```

:   **Description copied from class: `Simulator`**
:   Initializes the algorithm:

    - set t=0
    - reset the `AmountManager`
    - recalculate the propensities

    Gets called at the very beginning of `start`

    :   **Overrides:**: `initialize` in class `Simulator`

---


### reinitialize

```
public void reinitialize()
```

:   **Description copied from class: `Simulator`**
:   Reset propensities when a event has been executed.

    :   **Specified by:**: `reinitialize` in class `Simulator`

---


### performStep

```
public void performStep(SimulationController control)
```

:   **Description copied from class: `Simulator`**
:   Performs one simulation step. Between steps the terminating condition is checked.
    The simulators controller is passed, if an extending class wants to check it
    within one step. Implementing algorithms cannot be sure that the propensities
    are correct at the beginning of a step (e.g. if the volume changed). They should
    override `Simulator.setAmount(int, long)` and `Simulator.setVolume(double)`
    if they need correct values!

    :   **Specified by:**: `performStep` in class `Simulator`
    :   **Parameters:**: `control` - the simulators controller

---


### directMCTau

```
protected double directMCTau(double sum)
```

:   obtains a random (but following a specific distribution) timestep as described by the
    direct method in chapter 5A page 417ff

    :   **Parameters:**: `sum` - sum of the propensities **Returns:**: tau

---


### setVolume

```
public void setVolume(double volume)
```

:   **Description copied from class: `Simulator`**
:   Sets the volume of the reaction network.

    :   **Overrides:**: `setVolume` in class `Simulator`
    :   **Parameters:**: `volume` - the volume

---


### setAmount

```
public void setAmount(int species,
                      long amount)
```

:   **Description copied from class: `Simulator`**
:   Sets the amount of the given species. Propensities have to be recalculated!

    :   **Overrides:**: `setAmount` in class `Simulator`
    :   **Parameters:**: `species` - species index: `amount` - amount of species **See Also:**: `AmountManager.setAmount(int, long)`

---


### getName

```
public String getName()
```

:   **Description copied from class: `Simulator`**
:   Gets the name of the algorithm.

    :   **Specified by:**: `getName` in class `Simulator`
    :   **Returns:**: name of the algorithm

---


### isEfficientlyAdaptSum

```
public boolean isEfficientlyAdaptSum()
```

---


### setEfficientlyAdaptSum

```
public void setEfficientlyAdaptSum(boolean efficientlyAdaptSum)
```


---


|  |  |  |
| --- | --- | --- |
| **Overview** \| **Package** \| **Class** \| **Use** \| **Tree** \| **Deprecated** \| **Index** \| **Help** | |  |
| **PREV CLASS**   **NEXT CLASS** | **FRAMES**    **NO FRAMES**     **All Classes** |  |
| SUMMARY: NESTED \| FIELD \| CONSTR \| METHOD | DETAIL: FIELD \| CONSTR \| METHOD |  |


---
